# Effects of CYP3A4*22 polymorphism on trough concentration of tacrolimus in kidney transplantation: a systematic review and meta-analysis

**DOI:** 10.3389/fphar.2023.1201083

**Published:** 2023-07-26

**Authors:** Jung Sun Kim, Sunyoung Shim, Jeong Yee, Kyung Hee Choi, Hye Sun Gwak

**Affiliations:** ^1^ College of Pharmacy and Graduate School of Pharmaceutical Sciences, Ewha Womans University, Seoul, Republic of Korea; ^2^ College of Pharmacy, Gachon University, Incheon, Republic of Korea

**Keywords:** tacrolimus, CYP3A4*22, polymorphism, trough concentration, kidney transplantation

## Abstract

**Purpose:** Tacrolimus (Tac) is a widely used immunosuppressive agent in kidney transplantation. Cytochrome P450 (CYP), especially *CYP3A4* enzymes are responsible for the metabolism of drugs. However, the correlation between plasma Tac concentration and CYP3A4*22 gene variants is controversial. This meta-analysis aims to evaluate the association between CYP3A4*22 polymorphism and the dose-adjusted trough concentration (C_0_/D) of Tac in adult kidney transplant patients.

**Methods:** We conducted a literature review for qualifying studies using the PubMed, Web of Science, and Embase databases until July 2023. For the continuous variables (C_0_/D and daily dose), mean difference (MD) and corresponding 95% confidence intervals (CIs) were calculated to evaluate the association between the *CYP3A4* ^*^ *22* and Tac pharmacokinetics. We performed an additional analysis on the relationship of CYP3A5*3 with Tac PKs and analyzed the effects of CYP3A4*22 in CYP3A5 non-expressers.

**Results:** Overall, eight eligible studies with 2,683 renal transplant recipients were included in this meta-analysis. The CYP3A4*22 allele was significantly associated with a higher C_0_/D (MD 0.57 ng/mL/mg (95% CI: 0.28 to 0.86; *p* = 0.0001) and lower mean daily dose requirement (MD -2.02 mg/day, 95% CI: −2.55 to −1.50; *p* < 0.00001). An additional meta-analysis demonstrated that carrying the CYP3A5*3 polymorphism greatly impacted Tac blood concentration. From the result with CYP3A5 non-expressers, CYP3A4*22 showed significant effects on the Tac C_0_/D and dose requirement even after adjusting the effect of CYP3A5*3.

**Conclusion:** Patients with CYP3A4*22 allele showed significantly higher plasma C_0_/D of Tac and required lower daily dose to achieve the therapeutic trough level after kidney transplantation. These findings of our meta-analysis may provide further evidence for the effects of genetic polymorphism in *CYP3A4* on the PKs of Tac, which will improve individualized treatment in a clinical setting.

## 1 Introduction

Tacrolimus (Tac) is a widely used maintenance immunosuppressive agent to prevent graft rejection in kidney transplantation. Tac suppresses T-cell activation by inhibiting the calcineurin activity and exhibits excellent graft survival with a low incidence of rejection (Shapiro et al., 1999; Hamawy, 2003). However, its narrow therapeutic index requires close monitoring of Tac concentration to maintain the level within an optimal range (Venkataramanan et al., 1995). A supratherapeutic level results in drug toxicity and infection while a subtherapeutic level can lead to allograft rejection (Robles-Piedras and González-López, 2009).

Tac is also characterized by its high inter-individual variability in its pharmacokinetics (PKs) (Venkataramanan et al., 1995). This makes it difficult to predict the trough concentration and determine the optimal dose. Moreover, hepatic dysfunction, age, sex, ethnicity, albumin concentration, and gene polymorphism affect the PKs of Tac (Staatz and Tett, 2004).

As Tac is a dual substrate of P-glycoprotein and cytochrome P450 (CYP) 3A4 and 3A5, genetic polymorphisms related to the expression of these proteins have been studied to explain the between-subject PK variability (Saeki et al., 1993; Dai et al., 2006; de Jonge et al., 2009). Among them, CYP3A5*3 (rs776746; 6986A>G) is the most significant genetic determinant of Tac PKs (Kuehl et al., 2001; Billing et al., 2017; Khan et al., 2020). This polymorphism is known to decrease the metabolic activity of the CYP3A5 enzyme. Several studies showed that patients with the CYP3A5*3/*3 variant exhibited a higher trough concentration and required a lower dose of Tac to achieve the target concentration than those with wild-type allele (Tang et al., 2011; Zong et al., 2017; Khan et al., 2020). According to pharmacogenetic-based dosing guidelines such as Clinical Pharmacogenetic Implementation Consortium (CPIC) and the Dutch Pharmacogenetic Working Group (DPWG), lower doses of Tac are recommended for CYP3A5 non-expressers than CYP3A5 expressers (Birdwell et al., 2015; KNMP, 2020).

CYP3A4 plays a significant role in the drug metabolism of numerous drugs (Wrighton et al., 2000; Danielson, 2002). Due to its wide variation in enzyme activity among the population, *CYP3A4* polymorphisms could influence the PKs and efficacy of related drugs (Shiraga et al., 1994; Macphee et al., 2002; Mulder et al., 2021). CYP3A4*22 (rs35599367; g.15389C>T), a novel variant of *CYP3A4*, has been reported to have low messenger RNA (mRNA) expression and low activity of CYP3A4; accordingly, its relationship with drug response has been widely studied (Wang et al., 2011; Elens et al., 2013). Especially for Tac, several studies also have analyzed the effects of CYP3A4*22 on its PKs (Tavira et al., 2013; De Jonge et al., 2015; Lloberas et al., 2017). However, the results are still controversial, and a meta-analysis on the topic has not been conducted yet. Therefore, this meta-analysis aims to elucidate the correlation between CYP3A4*22 polymorphism and Tac concentration in adult patients with renal transplantation.

## 2 Methods

### 2.1 Search strategy of literature

This meta-analysis followed the Preferred Reporting Items for Systematic Reviews and Meta-Analyses (PRISMA) guidelines (Moher et al., 2009). A comprehensive search was performed for articles published before 13 July of 2023 in PubMed, Web of Science, and Embase based on PICO elements (Supplementary Table S1). The search strategy by using the following keywords: (Transplantation) AND (Polymorphis* OR SNP* OR mutation* OR variant* OR genotyp* OR allele*) AND (Tacrolimus OR FK506) AND (CYP3A4*) (Supplementary Table S2).

### 2.2 Study selection

The eligible studies were selected in the analysis if they 1) were cohort studies; 2) involved the adult renal transplantation patients who took tacrolimus; 3) evaluated the association between CYP3A4*22 genotypes and trough concentrations (C_0_) of Tac at steady state; 4) adjusted C_0_ by daily dose; and 5) expressed data as the mean with standard deviation (SD) or the median with range.

The studies were excluded if they were 1) not original articles (e.g., conference abstracts, letters, or reviews); 2) *in vitro* or *in vivo* studies; or 3) were conducted on patients with a single dose of Tac. If there was possibility of data overlap among the studies, only the most recent and comprehensive data was included.

### 2.3 Data extraction

Two authors (JSK and SS) performed the initial screening independently using Endnote to exclude duplicate studies. Next, the list of studies was compared, and consensus was achieved through discussion. Subsequently, both reviewers (JSK and SS) independently assessed the titles and abstracts, excluding studies that did not meet the inclusion and exclusion criteria. Throughout this process, the reviewers ensured methodological consistency and error reduction in the extraction techniques. Then, both authors (JSK and SS) independently evaluated the full text of all relevant studies to determine their eligibility. All studies that did not meet the eligibility criteria during the second screening were documented, along with the reasons for their exclusion. In case of any disagreements on study selection, a consensus was reached through discussion with a third reviewer (JY). For each study, extracted data were as follows: first authors, publication years, study design, country, ethnic background, characteristics of participants (population size, age, and weight), immunosuppressive protocol, alleles studied, genotyping methods, measurement methods for the C_0_ of Tac, the allele frequency of CYP3A4*22, dose-adjusted trough concentration (C_0_/D), and the daily dose of Tac according to post-transplantation period.

C_0_/D was calculated by the plasma trough concentration (ng/mL) of Tac divided by daily dose (mg), expressed as ng/mL per mg (Schütte-Nütgen et al., 2019). For continuous data, the mean and SD were extracted. For the studies providing data in the median with range, the method of Hozo et al. (2005) was used to estimate the mean and SD.

### 2.4 Quality assessment

The Newcastle-Ottawa Scale (NOS) system was adopted to rate the quality of the evidence. The total score of NOS ranges from 0 to 9; 0–4 points were assigned for the selection of the population, 0–2 points for comparability, and 0–3 points for the outcomes. For comparability, 1 point each was awarded if studies matched or adjusted with the age or other known risk factors.

### 2.5 Statistical analysis

For the continuous variables (C_0_/D and daily dose), mean difference (MD) and corresponding 95% confidence intervals (CIs) were calculated to evaluate the association between the CYP3A4*22 and Tac PKs.

All analyses were conducted using Review Manager (RevMan) version 5.4 (The Cochrane Collaboration, Copenhagen, Denmark) and R software (version 3.6.0). A *p* < 0.05 was considered statistically significant. Heterogeneity was evaluated via chi-square test and *I* ^2^ statistic. An *I* ^2^ < 50% was considered low heterogeneity, whereas an *I* ^2^ ≥ 50% high heterogeneity. If a low level of heterogeneity was observed, the fixed-effect model (Mantel-Haenszel method) was used; if not, the random-effect model (DerSimonian-Laird method) was applied (Biondi-Zoccai et al., 2011). Begg’s test and Egger’s test were used to identify publication bias (Begg and Mazumdar, 1994; Egger et al., 1997).

Sensitivity analyses were conducted to evaluate the robustness of the results by omitting the factor to assess its influence on the overall estimate. The first sensitivity analysis was performed by excluding each post-transplantation period at a time sequentially, and another sensitivity analysis by omitting studies that scored lower than 7 on the NOS system. We performed an additional analysis on the relationship of CYP3A5*3 with Tac C_0_ and daily dose. In order to observe the independent influence of CYP3A4*22 while controlling for CYP3A5*3, we analyzed the effects of CYP3A4*22 in CYP3A5 non-expressers.

## 3 Results

**FIGURE 1:**
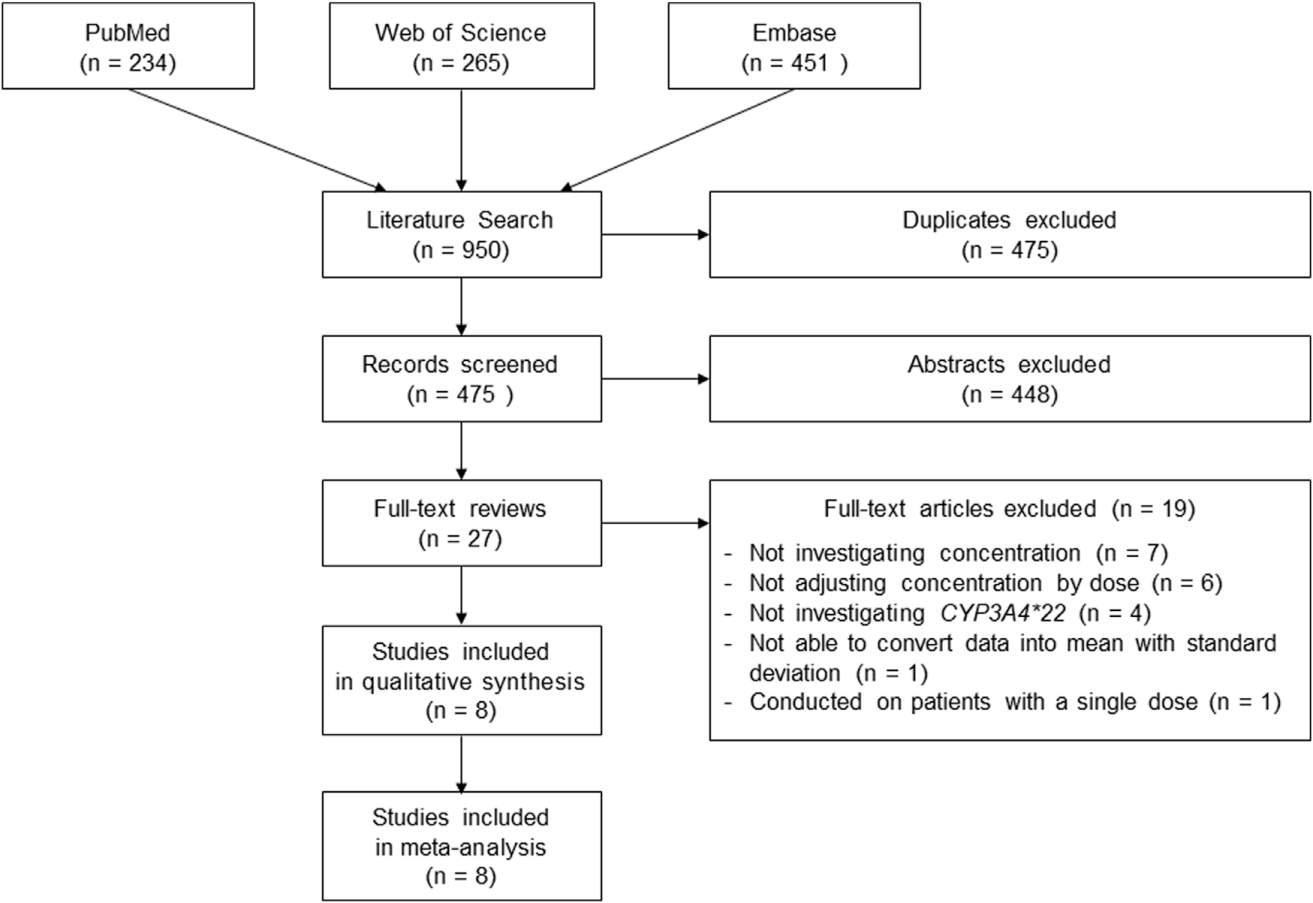
The selection process of the eligible studies in this meta-analysis.

A total of 950 studies were retrieved by the literature search and 475 duplicates were removed (Figure 1). After excluding 448 studies based on the titles and abstracts, 27 papers remained. We excluded 19 studies that did not investigate concentration (*n* = 7), did not adjust concentration by dose (*n* = 6), did not investigate CYP3A4*22 (*n* = 4), could not express data in mean with SD (n = 1), and administered a single dose (*n* = 1). Finally, eight cohort studies were selected, including data of 2,624 patients in the meta-analysis. The main characteristics of the eligible studies are presented in Table 1. All included studies were performed in hospital settings between 2013 and 2018. Most were performed on European patients. The mean age (years) and weight (kg) ranged from 48.6 to 54.4 and from 70.8 to 87.5, respectively. NOS ranged from 5 to 8.

**TABLE 1:** Characteristics of included studies.

First author (year)	Country	Ethnic background	Sample size (male %)	Age (years) (mean ± SD)	Weight (kg) (mean ± SD)	Immuno-suppressive protocol	Alleles studied	Genotyping method	Tac measurement method	CYP3A4*22 allele frequency (%)	NOS
Tavira et al. (2013)	Spain	Caucasians	206 (NA)	48.6 ± 13.6	NA	Tac, MMF, PD	CYP3A5*3	TaqMan	CLIA	4.9	7
							CYP3A4*1B				
Kuypers et al. (2014)	Belgium	Caucasians	246 (59.8)	53.0 ± 14.2	70.8 ± 13.5	Tac, MMF, mPD	CYP3A5*3	TaqMan	MEIA	NA	7
							POR*28				
Lunde et al. (2014)	Norway	Caucasians	123 (70.7)	48.8 ± 9.8	87.5 ± 19.1	Tac, MMF, steroids	CYP3A5*3	PCR equencing	CMIA	4.9	5
							POR*28				
							*PPARA* (rs4253728, rs4823613)				

De Jonge et al. (2015)	Belgium	Caucasians	80 (70.0)	54.6 ± 12.5	75.6 ± 14.7	Tac, MMF, mPD	CYP3A5*3	TaqMan	LC-MS	NA	8
Lloberas et al. (2017)	Spain	Caucasians	272 (65.8)	51.0 ± 15.0	69.6 ± 13.7	Tac, MMF, PD	CYP3A5*3	TaqMan	EMIT,	4.5	7
									LC-MS		
Madsen et al. (2017)	Denmark	Caucasians	52 (57.7)	49.3 ± 12.3	77.0 ± 20.0	Tac, MMF, steroids	CYP3A5*3	TaqMan	Immunoassay	2.9	6
							POR*28				
							*PPARA* (rs4253728)				

Vanhove et al. (2017)	Belgium	NA	279 (63.4)	53.0 ± 13.0	73.4 ± 15.2	Tac, MMF, mPD	CYP3A5*3	OpenArray	MEIA	3.4	7
Scheibner et al. (2018)	U.S.A	Caucasians	1,366 (63.3)	51.3 ± 13.0	83.7 ± 19.6	Tac, MMF	CYP3A5*3	NA	CLIA	5.6	7

CLIA, chemiluminescent immunoassay; CMIA, chemiluminescent microparticle immunoassay; EMIT, enzyme multiplied immunoassay technique; LC-MS, liquid chromatography-mass spectrometry; MEIA, microparticulate enzyme immunoassay; MMF, mycophenolate mofetil; mPD, methylprednisolone; NA, not available; NOS, Newcastle–Ottawa score; PCR, polymerase chain reaction; PD, prednisolone; SD, standard deviation; Tac, tacrolimus.

**FIGURE 2:**
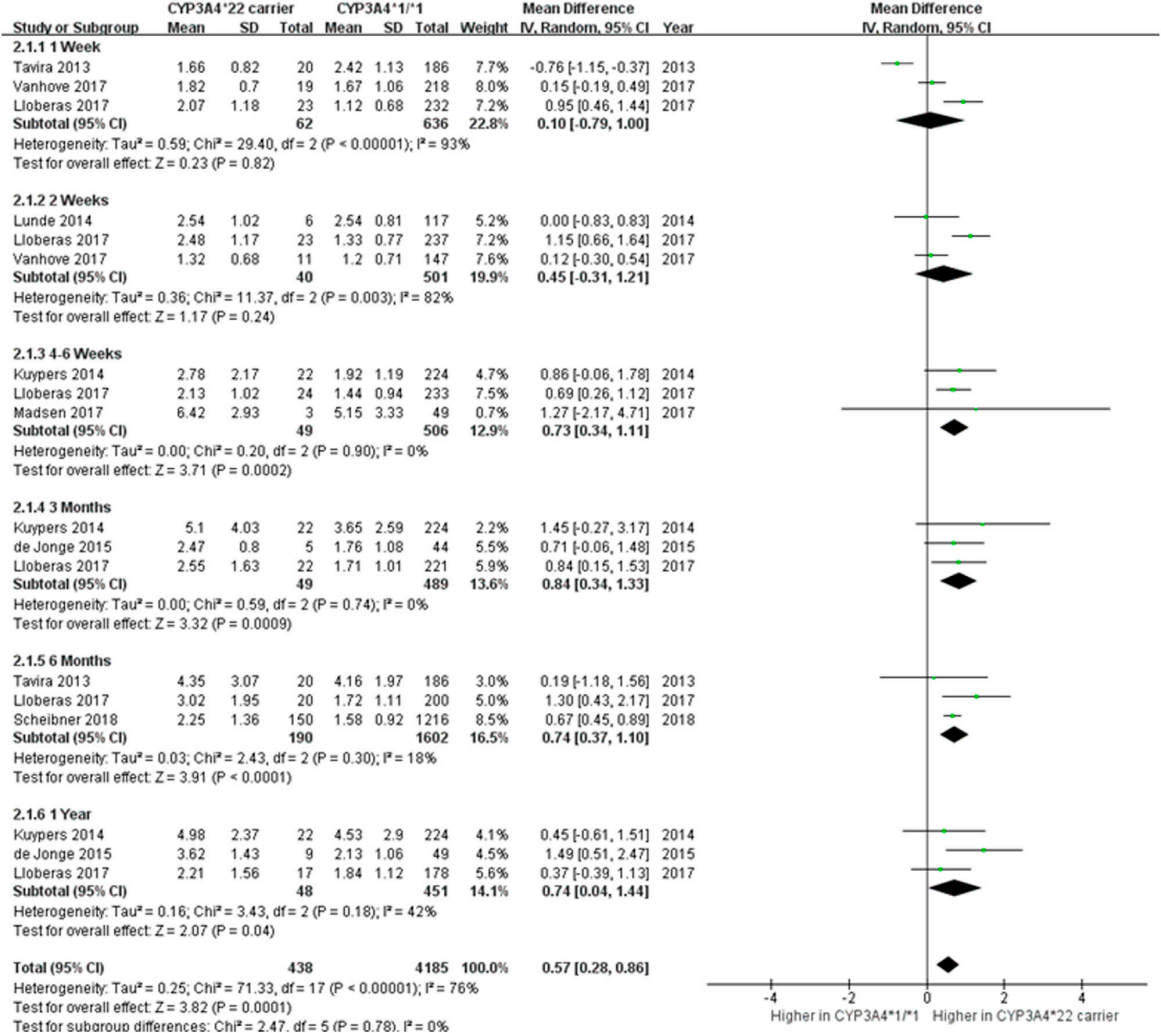
Forest plot showing the association between CYP3A4*22 polymorphism and C_0_/D.

The effects of the CYP3A4*22 genetic polymorphism on C_0_/D were evaluated by meta-analysis (Figure 2) (Tavira et al., 2013; Kuypers et al., 2014; Lunde et al., 2014; De Jonge et al., 2015; Lloberas et al., 2017; Madsen et al., 2017; Vanhove et al., 2017; Scheibner et al., 2018) Data from each study were analyzed by classifying post-transplant periods into 1 week, 2 weeks, 4–6 weeks, 3 months, 6 months, and 1 year. When data were combined in all study periods, the CYP3A4*22 carriers exhibited 0.57 ng/mL/mg higher C_0_/D than CYP3A4*1/*1 recipients (95% CI 0.28 to 0.86; *p* = 0.0001). Except for the first 2 weeks post-transplantation, statistically notable differences in the C_0_/D of Tac were detected according to CYP3A4*22 genotypes. Although substantial heterogeneity across the studies was found (*I* ^2^ = 76%, *p* < 0.00001), no subgroup difference was reported among the six different time periods (*p* = 0.78). Begg’s and Egger’s tests indicated no evidence of publication bias (*p* = 0.733 and *p* = 0.453, respectively).

**FIGURE 3:**
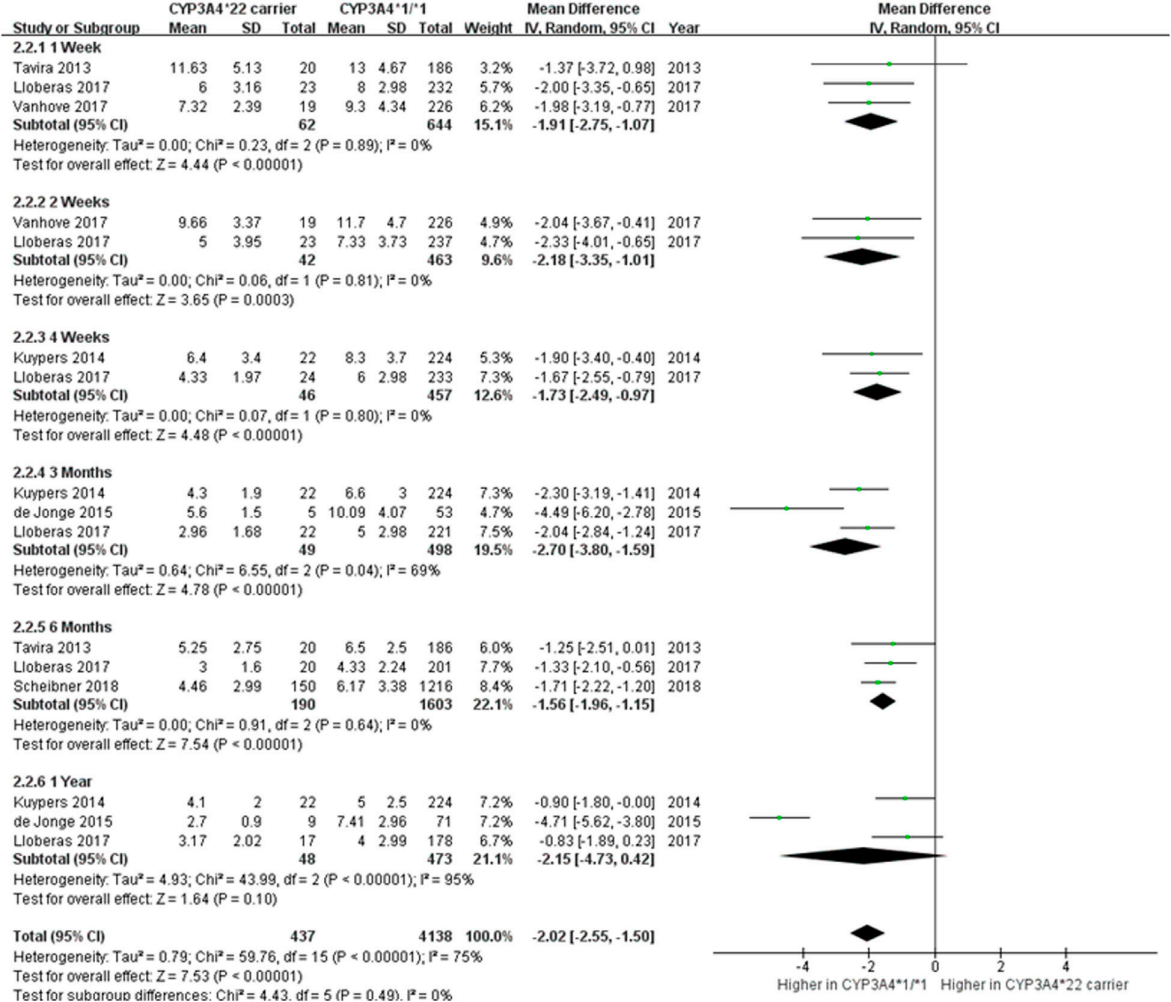
Forest plot showing the association between CYP3A4*22 polymorphism and a daily dose.

Six studies (Tavira et al., 2013; Kuypers et al., 2014; De Jonge et al., 2015; Lloberas et al., 2017; Vanhove et al., 2017; Scheibner et al., 2018) were analyzed to investigate the influence of the CYP3A4*22 variant on the daily dose of Tac (Figure 3). When data in all study periods were combined, CYP3A4*22 carriers required a 2.02 mg/day less dose to attain the optimal trough level than non-carriers (95% CI -2.55 to −1.50; *p* < 0.00001). Except for 1-year post-transplantation, significant differences in the daily dose were observed between CYP3A4*22 carriers and CYP3A4*1/*1 carriers. Similar to C_0_/D, there were substantial heterogeneity (*I* ^*2*^ = 75%, *p* < 0.00001) but no subgroup significant difference (*p* = 0.49). Results from Begg’s and Egger’s tests indicated no statistical evidence of publication bias (*p* = 0.177 and *p* = 0.568, respectively).

The first sensitivity analysis was performed by excluding each post-transplantation period at a time (Supplementary Table S3). In the sensitivity analysis of C_0_/D, heterogeneity was mitigated when data measured in the first week after transplantation (Biondi-Zoccai et al., 2011; Tavira et al., 2013; Lloberas et al., 2017) were excluded (*I* ^*2*^ *=* 30%, *p =* 0.13). The results of the dose requirement showed an MD range of −2.19 to −1.85 mg/day with an *I* ^2^ range of 15%–80%. For dose requirements, heterogeneity was greatly reduced when data measured 1 year after transplantation (Moher et al., 2009; De Jonge et al., 2015; Lloberas et al., 2017) were omitted (*I* ^*2*^ = 15%, *p =* 0.30). Another sensitivity analysis was performed with the studies that scored 7 or higher on the NOS system (Supplementary Table S4). The MD of C_0_/D was 0.60 ng/mL/mg, which was comparable to the main result.

As Tac is a substrate of CYP3A5, we performed an additional meta-analysis of the relationship between CYP3A5*3 and Tac PKs in the same cohorts. The CYP3A5*3/*3 carriers exhibited 1.23 ng/mL/mg higher C_0_/D than CYP3A5*1 carriers (95% CI 1.06 to 1.41, *p* < 0.00001; Supplementary Figure S1A). In order to attain the optimal trough level, CYP3A5*3/*3 carriers required 4.96 mg/day less dose than patients with CYP3A5*1 allele (95% CI −5.91 to −4.00, *p* < 0.00001; Supplementary Figure S1B).

**FIGURE 4:**
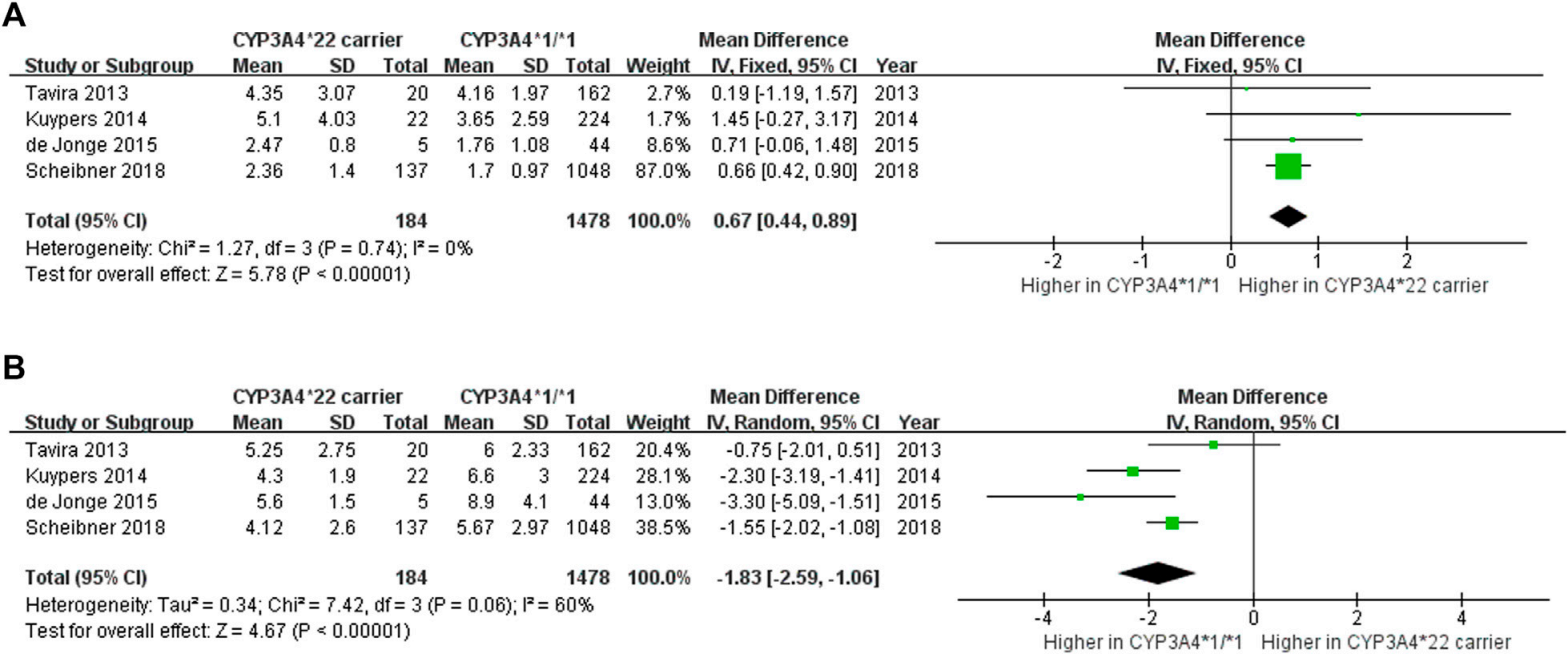
Forest plot showing the association between CYP3A4*22 polymorphism and **(A)** C_0_/D **(B)** daily dose in CYP3A5 non-expressers.

To further investigate the independent effect of CYP3A4*22 while adjusting for CYP3A5*3, we analyzed the effects of CYP3A4*22 in CYP3A5 non-expressers. Four studies (Egger et al., 1997; Moher et al., 2009; Tavira et al., 2013; De Jonge et al., 2015) elucidated the impact of the CYP3A4*22 genotype on C_0_/D and the dose requirement of Tac in CYP3A5 non-expressers. The evaluation of outcomes occurred within 3 to 6 months after kidney transplantation. When the effect of CYP3A5 was adjusted, the C_0_/D of CYP3A4*22 carriers was 0.67 ng/mL/mg higher (95% CI 0.44 to 0.89, *p* < 0.00001; Figure 4A) and dose requirement was 1.83 mg/day lower (95% CI − 2.59 to −1.06, *p* < 0.00001; Figure 4B) than patients with CYP3A4*1/*1. Therefore, the significant effect of CYP3A4*22 on C_0_/D and the dose requirement of Tac remained evident even after adjusting for CYP3A5*3.

## 4 Discussion

This is the first meta-analysis to evaluate the effects of the CYP3A4*22 variants on C_0_/D and the dose of Tac in adult renal transplant patients. Compared to patients with CYP3A4*1/*1, CYP3A4*22 carriers tend to exhibit increased C_0_/D and require a lower dose of Tac. Considering that C_0_/D is considered as a surrogate marker to determine the Tac metabolism rate (Thölking et al., 2014), this finding implies that CYP3A4*22 carriers may have lower CYP3A4 activity than CYP3A4*1/*1 carriers, thereby leading to overexposure to Tac, especially from first 4 weeks to 1 year after transplantation.

CYP3A4*22, an intronic variant of *CYP3A4,* occurs when C is substituted with T in intron 6 (Wang et al., 2011). In both *in vitro* and *in vivo* studies, this variant was associated with increased production of a non-functional *CYP3A4* alternative splice variant with partial intron 6 retention (Wang and Sadee, 2016). This resulted in decreased functional mRNA and protein production compared to the wild-type (Wang et al., 2011; Klein et al., 2012). Hence, it can be speculated that those with CYP3A4*22 may have lower CYP3A4 enzymatic activity and exhibit higher plasma concentration, which can lead to drug-induced toxicities.

In line with our results, several clinical studies showed that CYP3A4*22 was related to decreased metabolism and increased exposure to CYP3A substrate drugs. For example, CYP3A4*22 carriers showed 20% higher simvastatin plasma concentrations and 58% higher plasma concentration of simvastatin acid (Tsamandouras et al., 2014; Luzum et al., 2015). Similarly, CYP3A4*22 carriers showed a 2.5-fold concentration and 1.7-fold higher C_0_/D of quetiapine (van der Weide and van der Weide, 2014). For cyclosporine, another immunosuppressive agent, CYP3A4*22 was associated with increased concentration by 50% and decreased clearance by 15% (Lunde et al., 2014; Moes et al., 2014).

CYP3A5*3 is one of the most significant genetic determinants of Tac PKs (Kuehl et al., 2001; Billing et al., 2017; Khan et al., 2020). An additional meta-analysis demonstrated that carrying the CYP3A5*3 polymorphism greatly impacted Tac blood concentration. From the result with CYP3A5 non-expressers, CYP3A4*22 showed significant effects on the Tac trough concentration and dose requirement. Furthermore, there was no linkage disequilibrium between CYP3A5*3 and CYP3A4*22 reported in the included studies (Moher et al., 2009; De Jonge et al., 2015) and GBR/FIN populations of the 1000 Genomes Project (r^2^ = 0.004). This finding indicates that the CYP3A4*22 and CYP3A5*3 polymorphisms are independently associated with Tac exposure.

This meta-analysis revealed a significant degree of heterogeneity among the included studies. Statistical heterogeneity can be attributed to the small number of included studies and the wide range of sample sizes, varying from 52 to 1,366 patients. Also, some factors that can potentially contribute to clinical heterogeneity, including variances in analytic methods and target trough concentration. In the sensitivity analysis of C_0_/D, the heterogeneity was reduced when data collected within the first week after transplantation was excluded. This suggests that the observed heterogeneity may be attributed to the early post-transplant period, which is characterized by the insufficient function of the transplanted graft.

This study has several limitations. First, all included studies were conducted in European populations. This was because CYP3A4*22 is rarely found in African or Asian descent, whereas the allele frequency of CYP34*22 is approximately 8% in Caucasians (Okubo et al., 2013). Second, there is considerable heterogeneity in the clinical setting, such as immunosuppressive protocol, target trough level, and comorbidities. Lastly, confounding factors that could affect the PKs of Tac, including age, body weight, and co-medication were not adjusted.

## 5 Conclusion

CYP3A4*22 allele carriers showed significantly higher plasma C_0_/D of Tac and required a lower daily dose to achieve the therapeutic trough level after kidney transplantation. These findings of our meta-analysis may provide further evidence for the effects of genetic polymorphism in *CYP3A4* on the PKs of Tac, which will improve individualized treatment in a clinical setting.

## Data Availability

The original contributions presented in the study are included in the article/Supplementary Material, further inquiries can be directed to the corresponding author.
