# Supplementary material for: Effects of CYP3A4*22 polymorphism on trough concentration of tacrolimus in kidney transplantation: a systematic review and meta-analysis
Source: Front Pharmacol. 2023 Jul 26;14:1201083. doi: 10.3389/fphar.2023.1201083 (PMC10409991; doi:10.3389/fphar.2023.1201083)

Supplementary Table 1. PICO elements

| PICO elements | Description |
| --- | --- |
| Population | Kidney transplant recipients |
| Intervention | *CYP3A4*22* (rs35599367) C>T variant allele carriers |
| Comparator | *CYP3A4*22* (rs35599367) C>T variant allele non-carriers |
| Outcomes | Tacrolimus trough concentration, dose requirement |

Supplementary Table 2. Search strategy

| No | Search term | Pubmed | Web of science | Embase |
| --- | --- | --- | --- | --- |
| #1 | Transplantation | 1,000,043 | 640,905 | 1,542,295 |
| #2 | (Polymorphi*) OR (SNP*) OR (mutation*) OR (variant*) OR (genotyp*) OR (allele*) | 1,890,238 | 2,063,291 | 2,536,899 |
| #3 | #1 AND #2 | 50,890 | 33,349 | 103,219 |
| #4 | (Tacrolimus) OR (FK506) | 30,094 | 32,181 | 103,599 |
| #5 | #3 AND #4 | 1,290 | 1,593 | 6,124 |
| #6 | CYP3A4* | 11,949 | 12,766 | 16,719 |
| #7 | #5 AND #6 | 234 | 265 | 451 |

Supplementary Table 3. Sensitivity analysis on post-transplantation periods.

| Excluded period | Heterogeneity I^2^ (%) | Statistical model | Mean difference  [95% CI] |
| --- | --- | --- | --- |
| Dose-adjusted trough concentration (C_0_/dose, ng/mL/mg) | | | |
| None | 76 | Random | 0.57 [0.28, 0.86] |
| 1 Week | 30 | Fixed | 0.68 [0.47, 0.89] |
| 2 Weeks | 77 | Random | 0.60 [0.26, 0.93] |
| 4-6 Weeks | 80 | Random | 0.54 [0.21, 0.87] |
| 3 Months | 80 | Random | 0.52 [0.20, 0.84] |
| 6 Months | 78 | Random | 0.54 [0.19, 0.89] |
| 1 Year | 79 | Random | 0.54 [0.22, 0.86] |
| Daily dose requirement (mg/day) | | | |
| None | 75 | Random | -2.02 [-2.55, -1.50] |
| 1 Week | 80 | Random | -2.06 [-2.67, -1.45] |
| 2 Weeks | 78 | Random | -2.01 [-2.58, -1.44] |
| 4 Weeks | 78 | Random | -2.06 [-2.66, -1.47] |
| 3 Months | 76 | Random | -1.85 [-2.46, -1.25] |
| 6 Months | 78 | Random | -2.19 [-2.87, -1.51] |
| 1 Year | 15 | Fixed | -1.86 [-2.13, -1.58] |

CI: confidence interval

Supplementary Table 4. Sensitivity analysis on quality score of included studies.

| Excluded study | Heterogeneity I^2^ (%) | Statistical model | Mean difference  [95% CI] |
| --- | --- | --- | --- |
| None | 76 | Random | 0.57 [0.28, 0.86] |
| Lunde et al. 2014 and Madsen et al. 2017 | 79 | Random | 0.60 [0.29, 0.90] |

CI: confidence interval

Supplementary Figure 1. Forest plot showing the association between *CYP3A5*3* polymorphism and (a) C_0_/D (b) daily dose.

(a)


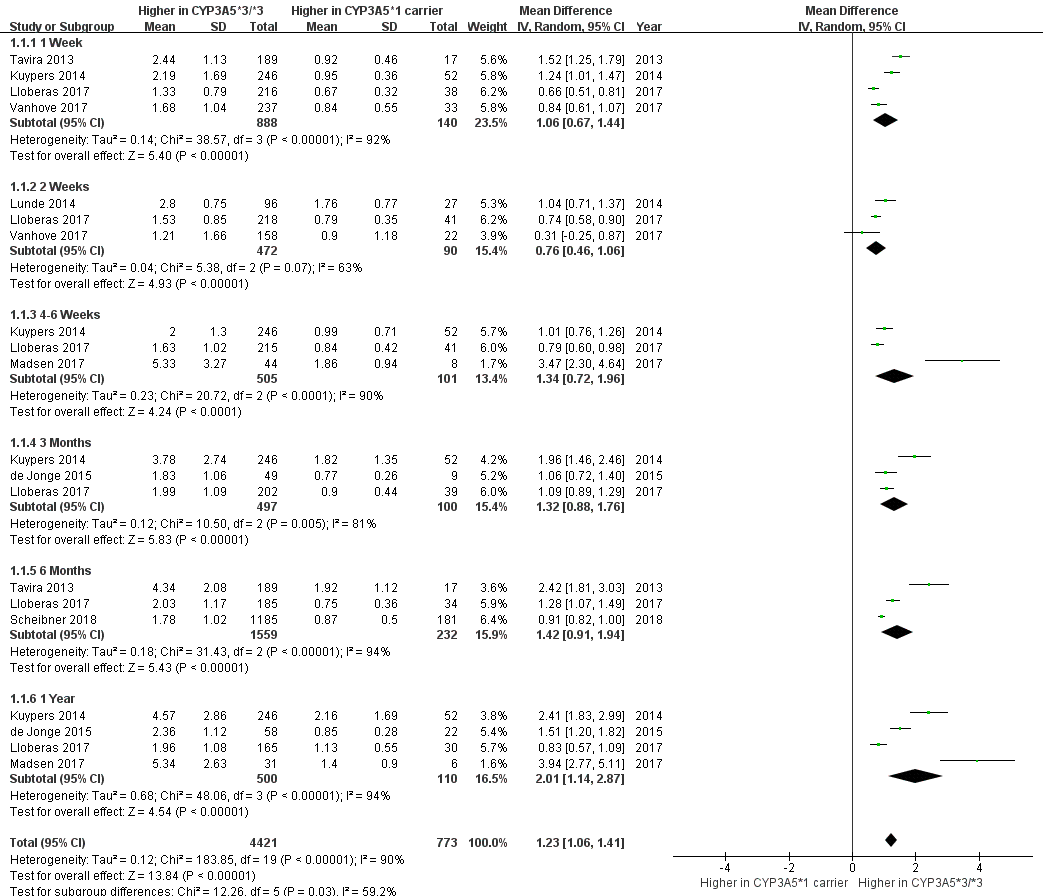


(b)


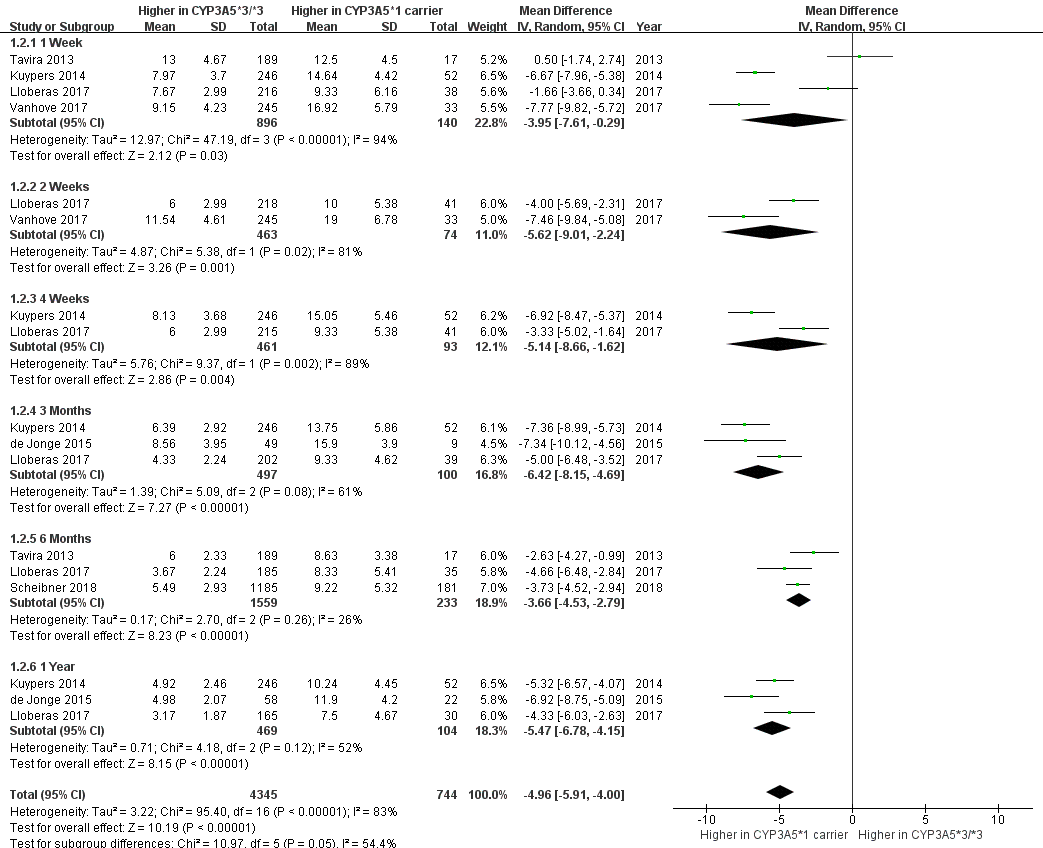

Supplement: Supplementary file 1 [file DataSheet1.docx]
